# Social class and gender patterning of insomnia symptoms and psychiatric distress: a 20-year prospective cohort study

**DOI:** 10.1186/1471-244X-14-152

**Published:** 2014-05-25

**Authors:** Michael J Green, Colin A Espie, Michael Benzeval

**Affiliations:** 1 MRC/CSO Social and Public Health Sciences Unit, University of Glasgow, 4 Lilybank Gardens, Glasgow, G12 8RZ, UK; 2 Nuffield Department of Clinical Neurosciences/Sleep & Circadian Neuroscience Institute, University of Oxford, Level 6, West Wing, John Radcliffe Hospital, Oxford, OX3 9DU, UK; 3 Institute of Social and Economic Research, University of Essex, Colchester, CO4 3SQ, UK

**Keywords:** Insomnia, Psychiatric distress, Longitudinal, Socioeconomic position, Gender

## Abstract

**Background:**

Psychiatric distress and insomnia symptoms exhibit similar patterning by gender and socioeconomic position. Prospective evidence indicates a bi-directional relationship between psychiatric distress and insomnia symptoms so similarities in social patterning may not be coincidental. Treatment for insomnia can also improve distress outcomes. We investigate the extent to which the prospective patterning of distress over 20 years is associated with insomnia symptoms over that period.

**Methods:**

999 respondents to the Twenty-07 Study had been followed for 20 years from approximately ages 36–57 (73.2% of the living baseline sample). Psychiatric distress was measured using the GHQ-12 at baseline and at 20-year follow-up. Gender and social class were ascertained at baseline. Insomnia symptoms were self-reported approximately every five years. Latent class analysis was used to classify patterns of insomnia symptoms over the 20 years. Structural Equation Models were used to assess how much of the social patterning of distress was associated with insomnia symptoms. Missing data was addressed with a combination of multiple-imputation and weighting.

**Results:**

Patterns of insomnia symptoms over 20 years were classified as either healthy, episodic, developing or chronic. Respondents from a manual social class were more likely to experience episodic, developing or chronic patterns than those from non-manual occupations but this was mostly explained by baseline psychiatric distress. People in manual occupations experiencing psychiatric distress however were particularly likely to experience chronic patterns of insomnia symptoms. Women were more likely to experience a developing pattern than men, independent of baseline distress. Psychiatric distress was more persistent over the 20 years for those in manual social classes and this effect disappeared when adjusting for insomnia symptoms. Irrespective of baseline symptoms, women, and especially those in a manual social class, were more likely than men to experience distress at age 57. This overall association for gender, but not the interaction with social class, was explained after adjusting for insomnia symptoms. Sensitivity analyses supported these findings.

**Conclusions:**

Gender and socioeconomic inequalities in psychiatric distress are strongly associated with inequalities in insomnia symptoms. Treatment of insomnia or measures to promote healthier sleeping may therefore help alleviate inequalities in psychiatric distress.

## Background

Anxiety and depression symptoms (or psychiatric distress) are more common for those in a disadvantaged socioeconomic position (SEP) [1-4] and inequalities widen with increasing age [5-8]. Gender inequalities, with higher levels of psychiatric distress among adult women, are also a common finding [9,10], and these too widen with age [11,12]. Gender differences are partially but not completely explained by female socioeconomic disadvantages [11]. Additionally, psychiatric distress predicts later insomnia symptoms (defined here as trouble initiating or maintaining sleep) [13,14], and, vice versa, insomnia predicts later psychiatric distress [14-18], even with adjustment for baseline or historic symptoms. This bi-directional evidence is suggestive of a positive feedback loop where each problem aggravates the other. Indeed, insomnia symptoms exhibit similar social patterning. A female propensity for insomnia symptoms, especially at older ages, has been established in a meta-analysis [19], and cross-sectional studies on population samples indicate that insomnia symptoms are more commonly experienced by those in a disadvantaged SEP [20,21]. Gender differences in insomnia symptoms are attenuated but not fully explained with adjustment for socioeconomic position [20,22,23]. Longitudinal evidence on insomnia is sparse but indicates that women have a propensity to develop insomnia symptoms in middle age and that those in a disadvantaged SEP are more likely to experience chronic, persistent insomnia symptoms than those more affluent [23]. The mutual association between insomnia symptoms and psychiatric distress may mean the similarities in patterning are not coincidental.

Since insomnia symptoms can come within the range of symptoms that indicate psychiatric distress, it is tempting perhaps to dismiss the social patterning of insomnia symptoms as an expression of inequalities in psychiatric distress. This may be true, but insomnia symptoms can lead to greater distress as well as vice versa. Indeed, trials have shown improved depression outcomes where concurrent insomnia is treated [24,25] and some have even called for trials of insomnia treatment to prevent depression [17,26]. If inequalities in psychiatric distress are even partially mediated by inequalities in insomnia symptoms then insomnia treatment might have the added benefit of reducing inequalities in psychiatric distress. With this in mind we investigated the extent to which long-term sleep patterns might explain subsequent inequalities in psychiatric distress, taking earlier distress into account. We focus on symptoms in mid-life as this is the life-stage where there is the most widening of socioeconomic inequalities in psychiatric distress [8], and where women are particularly likely to develop insomnia symptoms [23].

## Methods

### Sample and measures

The Twenty-07 Study [27] has followed people in three age cohorts – born around 1932, 1952, and 1972 - for 20 years. It has two samples: the regional sample, a two-stage stratified random sample of people living in an area of the West of Scotland centred on Glasgow (previously known as the Central Clydeside Conurbation), and the localities sample of people from two areas of the city of Glasgow. Baseline interviews were conducted in 1987/88 and there have been four follow-ups (1990/2; 1995/7; 2000/4; 2007/8). Ethical approval was gained for each wave from the NHS and/or Glasgow University Ethics Committees. The 1950s cohort aged from approximately 36 to 57 years during the study period, with a baseline sample size of 1,444 (response rate was 88.9%). The achieved sample has been shown to be representative of the general population of the sampled area [28]. This paper focuses on a sub-set of respondents from this cohort who participated in both the baseline and the final interview in 2007/08 (n = 999; 73.2% of the living baseline sample).

**Figure 1:**
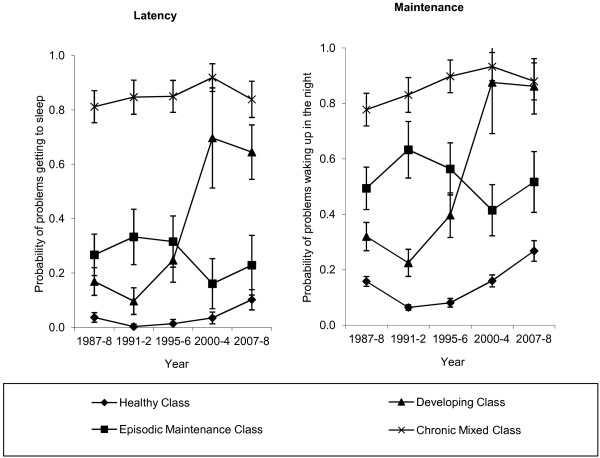
Latent class patterns of insomnia symptoms over 20 years.

Insomnia symptoms (latency and maintenance) were self-reported by the study respondents at each wave. For latency, in the first three interviews respondents were asked, “How often do you have trouble getting to sleep?” and in interviews four and five, “During the past month how often have you had trouble sleeping because you cannot get to sleep within 30 minutes?”. For maintenance, in the first three interviews respondents were asked “How often are you bothered by waking earlier than you would like to, or by waking up in the middle of the night?” and in interviews four and five: “During the past month how often have you had trouble sleeping because you wake up in the middle of the night or early morning?”. Responses were coded to indicate problems occurring at least weekly. A latent class variable was derived from the responses at all five interviews which categorised individual longitudinal patterns of insomnia symptoms into the four groups depicted in Figure 1 (for further details, including a consideration of the change in question wording, see Green et al. 2012) [23]. The four groups included: a *Healthy* pattern with low levels of symptoms across the 20 years; a pattern with episodic problems maintaining sleep, but relatively few problems initiating sleep (*Episodic Maintenance*); a pattern where insomnia symptoms were not present initially but developed later (*Developing*); and a chronic pattern with a high likelihood of problems both maintaining and initiating sleep across the 20 years (*Chronic Mixed*). This latent class variable constitutes the measure of insomnia symptoms used in this study and represents an individual’s pattern of symptoms across the 20 year study period rather than at any single point in time. Classification of individuals into classes was reasonably definitive (entropy statistic: 0.68), but not certain.

Psychiatric distress was measured at baseline and at the final interview using the 12-item General Health Questionnaire (GHQ) [29]. Scores of 2 or more were coded as indicating probable psychiatric morbidity. Gender was coded 0 for males and 1 for females. SEP was measured with baseline household occupational class, coded according to the Registrar General’s 1980 classification [30], using the higher status occupation from couple households. If neither the respondent nor their partner had a current occupation then the most recent previous occupations were used. This was dichotomised with 0 for non-manual (I through to III non-manual) and 1 for manual (III manual through to V). Medications were self-reported and then assigned British National Formulary codes by medically-trained coders.

### Statistical analyses

**Figure 2:**
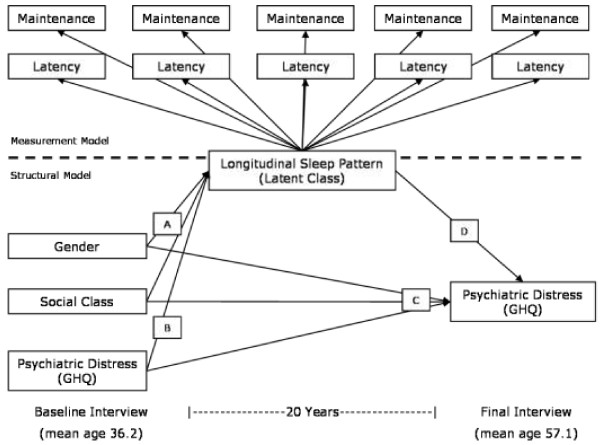
Diagram of analysis model.

Figure 2 illustrates the structural equation model used. A structural equation model can be thought of as a combination of measurement models, in which latent variables are defined, and a structural model describing relationships between those latent variables and other observed variables. The measurement model used here is the latent class variable representing longitudinal patterns of insomnia symptoms. This is estimated jointly with the wider structural equation model, in order to account for the fact that latent class membership is uncertain [31].

The structural part of the model relates this latent class variable to other observed variables, proceeding through four main stages. Initially, gender and baseline social class are used as predictors of latent class membership (model A). This establishes the gender and social class patterning of the latent classes, replicating our previous findings [23]. Adjustment is then made for baseline psychiatric distress (model B) in order to see how much of the gender and social class patterning of insomnia symptoms can be accounted for by initial distress.

Prospective associations between gender and baseline social class and psychiatric distress at age 57 are then estimated with adjustment for baseline psychiatric distress (model C). Finally, psychiatric distress at age 57 is additionally adjusted for latent class membership (model D), in order to see how much of the prospective associations with gender and social class are mediated via long-term sleeping patterns. Interactions between gender, social class and psychiatric distress were tested at all stages and were retained if they were significant at the P < 0.05 level at any stage. Adding variables to a latent class model can potentially alter class membership assignments [31] so parameters were fixed to enhance stability across model stages. Class assignments were generally more than 95% stable across different models, and the few who did switch classes between model stages tended to be those who were uncertainly assigned anyway (i.e. their responses didn’t fit well with any of the four patterns).

Analyses were conducted in Mplus version 7 [32], using maximum likelihood estimation with robust standard errors to account for non-normality of data and clustered sampling. Results are presented as odds ratios (OR) with 95% confidence intervals (95% CI). Considering attrition, the analysis sample was weighted to the living baseline sample [33]. However, of the 999 who participated in the fifth interview, only 859 (86%) had full data for all the variables in the structural part of the model. Multiple imputation with an unrestricted model [34] of all the analysis variables (including the weights) was therefore used to estimate missing values for the structural part of the model. It was not necessary to impute data for the measurement part of the model as respondents could still be assigned to latent classes based on their observed responses. Additionally, data on GHQ scores and anti-depressant medication from intervening interviews were included where available to strengthen the imputation model [35]. Fifteen imputed datasets were created and analysis results were averaged across these datasets [36].

Three further sensitivity analyses were conducted, essentially repeating the main analyses (including the imputation of missing values) with different definitions of psychiatric distress. Two further binary definitions were used. First, as one of the GHQ items (‘Have you recently lost much sleep over worry?’) relates to sleeping difficulties the analysis was repeated with this item excluded from the calculation of the GHQ scores. This tests whether observed associations are the result of measurement overlap. Second, as some respondents might have had low GHQ scores because they were taking anti-depressant medication to manage their symptoms the analysis was repeated with psychiatric distress indicated by either a high GHQ score (2+), or reporting taking anti-depressant medication. Finally, the analyses were also repeated using the continuous GHQ scores, to see if results differed with greater sensitivity to the severity of psychiatric distress being experienced.

## Results

Descriptive characteristics of the sample (before weighting and imputation) are displayed in Table 1. Psychiatric distress was present in a little more than a quarter of the sample at both ages 36 and 57. The *Healthy* sleep pattern was most common, followed by the *Developing* and *Episodic Maintenance* patterns, whilst *Chronic Mixed* was least common.

**Table 1:** Descriptive data for the analysis variables

**Sample characteristics (n = 999)**		**N (%)**
Gender	Male	457 (45.7)
	Female	542 (54.3)
Social class at age 36	Non-manual	686 (68.7)
	Manual	304 (30.4)
	Missing	9 (0.9)
GHQ (2+) at age 36	No	593 (59.4)
	Yes	303 (30.3)
	Missing	103 (10.3)
GHQ (2+) at age 57	No	706 (70.7)
	Yes	255 (25.5)
	Missing	38 (3.8)
Most likely sleep class	Healthy	464 (46.4)
	Episodic maintenance	164 (16.4)
	Developing	250 (25.0)
	Chronic Mixed	112 (11.2)
	Missing	9 (0.9)

Table 2 displays the results from the first two stages. In model A, as found previously [23], relative to those with *Healthy* sleep patterns, women were more likely to be in the *Developing* class than men. In reference to the *Healthy* class, those from manual social classes were more likely to be in any of the symptomatic sleep classes than those from non-manual occupations, though this relationship was weak (p = 0.097) for the *Episodic Maintenance* class, and strongest for the *Chronic Mixed* class. When baseline psychiatric distress was adjusted for in model B, the associations between sleep classes and gender were largely unaffected but social class was no longer significantly associated with membership in the *Episodic Maintenance* or *Chronic Mixed* classes and the association weakened for the *Developing* class (p = 0.051). Respondents with baseline psychiatric distress were generally more likely to be members of symptomatic sleep classes than to be healthy sleepers. However, social class was still important for sleep as an interaction was found whereby those in a manual social class with high baseline GHQ scores were especially likely to be in the *Chronic Mixed* class (rather than the *Healthy* class). All three sensitivity analyses replicated these findings (see Additional file 1).

**Table 2:** Odds ratios for sleep class membership with and without adjustment for baseline psychiatric distress

	**Model A: Predicting sleep**		**Model B: Predicting sleep with adjustment for psychiatric distress**	
	**OR**	**95% CI**	**OR**	**95% CI**
*Episodic maintenance class* (*ref*: *Healthy*)				
Female gender	0.85	(0.49-1.47)	0.81	(0.45-1.46)
Manual social class	1.60*	(0.92-2.78)	1.55	(0.65-3.69)
GHQ (2+) at age 36			3.57**	(1.82-7.02)
Social class and GHQ interaction			1.26	(0.31-5.10)
*Developing class* (*ref*: *Healthy*)				
Female gender	4.20**	(2.68-6.60)	4.02**	(2.52-6.42)
Manual social class	1.86**	(1.27-2.72)	1.73*	(1.00-2.99)
GHQ (2+) at age 36			2.32**	(1.26-4.28)
Social class and GHQ interaction			1.54	(0.40-5.97)
*Chronic mixed class* (*ref*: *Healthy*)				
Female gender	1.36	(0.78-2.37)	1.14	(0.63-2.07)
Manual social class	3.34**	(1.69-6.57)	0.98	(0.31-3.08)
GHQ (2+) at age 36			3.26**	(1.43-7.45)
Social class and GHQ interaction			9.57**	(2.55-35.95)

*p < 0.1.

**p < 0.05.

The results from models predicting psychiatric distress at age 57 are displayed in Table 3. In model C, without adjustment for sleep class membership, women and those with high baseline GHQ scores were more likely to have high GHQ scores at age 57. Both of these effects were stronger for those in manual social classes relative to their non-manual counter-parts, though the gender by social class interaction was statistically weak (p = 0.072). Model D adjusted for sleep class membership. All of the symptomatic sleep classes, but especially the *Chronic Mixed* class, were associated with higher odds of psychiatric distress at age 57 than in the *Healthy* sleep class. The gender and baseline GHQ effects were attenuated, but only the gender difference became non-significant. The interaction between social class and baseline GHQ was also attenuated into non-significance, but the interaction between social class and gender was actually stronger with adjustment for insomnia symptoms, passing the p < 0.05 level.

**Table 3:** **Odds ratios for psychiatric distress at age 57**, **with and without adjustment for insomnia symptoms**

	**Model C: Predicting distress**		**Model D: Predicting distress with adjustment for sleep**	
	**OR**	**95% CI**	**OR**	**95% CI**
Female gender (ref: Male)	1.45**	(1.01-2.09)	1.24	(0.79-1.97)
Manual social class (ref: Non-manual)	1.03	(0.57-1.87)	0.92	(0.47-1.78)
GHQ (2+) at age 36 (ref: <2)	2.20**	(1.55-3.13)	1.69**	(1.16-2.47)
Gender and Social class interaction	2.00*	(0.96-4.14)	2.16**	(1.05-4.46)
Social class and GHQ Interaction	2.19**	(1.08-4.45)	1.51	(0.71-3.20)
Episodic maintenance class (ref: Healthy)			2.90**	(1.35-6.25)
Developing class (ref: Healthy)			4.09**	(2.36-7.09)
Chronic mixed class (ref: Healthy)			9.23**	(5.10-16.68)

*p < 0.1.

**p < 0.05.

The sensitivity analyses for models C and D produced broadly consistent findings (details in Additional file 1). The *Episodic Maintenance*, *Developing*, and *Chronic Mixed* classes (relative to the *Healthy* class) were associated respectively with mean differences of 1.04, 1.36 and 2.16 GHQ points at age 57 (standard errors were 0.30, 0.24 and 0.41), adjusting for the other variables in the model (including baseline GHQ scores).

## Discussion

By using 20 years of longitudinal data this paper goes further in demonstrating the inter-relatedness of insomnia and psychiatric distress than is possible with cross-sectional research or longitudinal research that only utilises cross-sectional (e.g. baseline) measures of insomnia symptoms. Even after adjustment for baseline psychiatric distress middle-aged women experienced greater odds of developing insomnia symptoms in late middle-age than men, and those who experienced psychiatric distress and socioeconomic disadvantage were more likely than others to be experiencing chronic insomnia symptoms. Psychiatric distress was more likely to recur or still be present 20 years later for those in socioeconomic disadvantage compared to those more affluent, but this association was reduced to non-significance when conditioning on intervening insomnia symptom trajectories. Irrespective of earlier psychiatric distress, women were more likely than men to experience psychiatric symptoms around age 57, though women from a manual social class were particularly disadvantaged in this respect. The greater likelihood of insomnia symptoms developing in middle age among women compared to men explained the overall association between gender and psychiatric distress at age 57, but not the extra disadvantages for women in a manual social class.

The findings concur with previous research showing that psychiatric distress and insomnia symptoms occur more frequently among women and those in socioeconomic disadvantage [2,3,19]. Cross-sectional evidence shows that sleep quality can mediate associations between socioeconomic disadvantage and poor mental health [37,38], but these findings show that this can be true over the long-term, up to 20 years, and that gender differences in mental health can also be mediated by sleeping problems. The findings agree with evidence that socioeconomic inequalities in psychiatric distress are at least in part due to symptoms being more persistent among the disadvantaged [3,39,40], and these results show that chronic insomnia symptoms are associated with socioeconomic inequalities in persistent distress. Research showing that more severe insomnia, including problems with both sleep maintenance and initiation, is associated with more severe psychiatric symptoms was also supported [41], and the findings add that this is particularly true for those who experienced long-term problems with both sleep maintenance and initiation.

It is clear from this study that insomnia symptoms and psychiatric distress are strongly associated with each other over the long-term, and that the social patterning of these problems is not independent. The propensity for those in socioeconomic disadvantage to experience persistent or recurring psychiatric distress is strongly associated with the propensity of these individuals for chronic insomnia symptoms, and the propensity for women to experience psychiatric distress in late-middle age is strongly associated with their propensity to develop insomnia symptoms around this time. Poor sleep appears to be characteristic of the type of psychiatric distress which is experienced more often by women and those in socioeconomic disadvantage. This suggests that treatment of insomnia has potential value for alleviating inequalities in psychiatric distress. Inequalities in psychiatric distress might also be helped by tackling the structural or societal constraints that make healthy sleep more difficult for particular groups [42]. The only element of the social patterning of psychiatric distress which was not strongly associated with insomnia symptoms in this study was the particular tendency for women in socioeconomic disadvantage to have high GHQ scores, and so such interventions would be limited in the extent to which they could alleviate this inequality.

Insomnia symptoms are thought to precipitate poor mental health under a diathesis-stress model [43] where the stress and opportunities for negative rumination associated with long, wakeful nights facilitate expression of a latent vulnerability to psychopathology [16,41]. Insomnia with short sleep duration can also impact on neuro-cognitive performance (e.g. attention switching, visual memory, processing speed) [44], and such performance deficits could be distressing to experience. Conversely, anxious, negative rumination may make it more difficult to sleep [16,41]. A diathesis-stress model is also often used to account for socioeconomic inequalities in distress [43], with the accumulation of stressful circumstances and low levels of coping resources associated with socioeconomic disadvantage explaining the higher levels of psychiatric morbidity, rather than differences in underlying vulnerability between social strata [45,46]. Socioeconomic disadvantage has additionally been posited as restricting the autonomy required to create ideal conditions for sleep [42]; recommendations such as not taking your problems to bed, for example, could be especially difficulty for those with more problems due to their disadvantaged circumstances, or those in lower status occupations may be more likely to experience disruptions to sleeping patterns from shift work. The inter-play of these processes could easily result in the findings reported here, which suggest a clustering of co-morbid persistent psychiatric distress and chronic insomnia symptoms among those in socioeconomic disadvantage.

As socioeconomic factors partially explain gender differences in psychiatric distress and insomnia symptoms [11,20,22], it is plausible that similar processes partially account for these differences. However, as in previous research, there were still independent effects of gender in this study even after conditioning on social class, and so further explanation is needed beyond gender inequities in the socioeconomic distribution. One hypothesis is that women experience additional stress from domestic and caring roles, over and above roles in employment or elsewhere, but studies have failed to show that this substantially accounts for gender differences in psychiatric distress or insomnia [22,47,48]. Female sex may also constitute a biological diathesis, with sex differences having been shown in a number of biological processes connected with depression [49]. This would lead to stronger gender differences where stress levels were also high, such as in a disadvantaged SEP, as was found in this study. A biological process uniquely experienced by women and particularly relevant to the age-group under study here is the menopausal transition, but evidence is mixed as to associations between menopause and depression or insomnia symptoms [50-52], and suggests that links could be accounted for by other life experiences common to women of this age in this cultural context such as the onset of chronic conditions, or children leaving home [53,54]. If some of these life experiences were more common amongst people living in socioeconomic disadvantage, then this could also explain the interaction with social class.

The findings support the idea of a positive feedback loop where insomnia and psychiatric distress aggravate each other [16,41]. There are nevertheless some limitations to the causal inferences that can be made, considering that the measure of sleep patterning includes indicators of insomnia symptoms at ages 36 and 57 (i.e. concurrent with the measures of psychiatric distress). The insomnia symptoms expressed in the *Chronic Mixed* or *Episodic Maintenance* patterns tended to be present at baseline and therefore probably began earlier so it is not clear whether they preceded or were preceded by psychiatric distress or socioeconomic disadvantage. Only with the *Developing* pattern is there evidence that earlier psychiatric distress predicts the later development of insomnia symptoms. Gender, taken here as an indication of biological sex, probably precedes insomnia symptoms and psychiatric distress (implying causal direction), both because biological sex is usually constant over the lifecourse, and because gender was primarily associated with symptoms that developed post-baseline. Symptomatic sleep patterns did predict distress at age 57, but were also predicted by distress at age 36, so the link may be associative rather than causal. Questions about causal direction between sleep and psychiatric distress have been addressed elsewhere, tending to show relationships in both directions [13-15,17,18,41], but the main focus here was on the social patterning. Additionally, considering the five year gaps between measurements, apparently persistent symptom patterns may represent recurring rather than persistent experiences. A further limitation is that people were only observed between ages 36 and 57; date from the USA suggests that the prevalence of sleep disturbance rises between ages 45–59 in men and women, but declines at older ages [55], so the symptoms observed in this cohort may not persist into later life. However, our previous research found higher levels of insomnia symptoms in the older cohort of the Twenty-07 study as they aged from 56–76 years [23].

## Conclusions

Overall this study demonstrates that the patterning of psychiatric distress and insomnia symptoms by gender and social class are strongly linked. Socioeconomic disadvantage is associated with a tendency to experience both chronic insomnia symptoms and persistent psychiatric distress, whilst women appear to be more likely than men to develop both insomnia symptoms and psychiatric distress in middle age. Recognising the association between inequalities in psychiatric distress and insomnia symptoms may help efforts to alleviate these inequalities.

## Abbreviations

SEP: Socioeconomic position; GHQ: General health questionnaire; OR: Odds ratio; CI: Confidence interval.

## Competing interests

MJG and MB declare that they have no competing interests. CAE declares that he has received payment for other work from Boots UK, Novartis, UCB pharma, and Constable & Robinson; has received grant funding from the NIH, the Chief Scientist Office in Scotland and Breast Cancer Research; and is a co-founder and shareholder of Sleepio Ltd.

## Authors’ contributions

MG designed the study, performed the statistical analysis and drafted the manuscript. CE and MB participated in the design of the study and helped to draft the manuscript. All authors read and approved the final manuscript.

## Pre-publication history

The pre-publication history for this paper can be accessed here:

http://www.biomedcentral.com/1471-244X/14/152/prepub

## Supplementary Material

Additional file 1 Sensitivity analyses. Click here for file
