# Supplementary material for: Social class and gender patterning of insomnia symptoms and psychiatric distress: a 20-year prospective cohort study
Source: BMC Psychiatry. 2014 May 25;14:152. doi: 10.1186/1471-244X-14-152 (PMC4041899; doi:10.1186/1471-244X-14-152)
Supplement: Additional file 1 — Sensitivity analyses. [file 1471-244X-14-152-S1.docx]

**Appendix**

Tables A1-A3 show ORs and CIs from models A and B in each of the three sensitivity analyses. The main findings are replicated in each instance.

Table A4 shows results from models C and D when the sleep item was excluded from the calculation of the GHQ scores. The association with gender and the interaction between social class and GHQ in model C were weaker (p=0.062 and p=0.071 respectively), but model D was replicated. This indicates those associations may have been partially but not entirely due to measurement overlap between sleep and psychiatric distress, but if anything this underlines the finding that insomnia symptoms are an intrinsic part of the psychiatric distress being experienced unequally by women and those in socioeconomic disadvantage.

Table A5 shows findings when anti-depressant medication was added to the definition of psychiatric distress. Only nine extra cases were identified at baseline, but 52 cases were added at age 57, perhaps reflecting a temporal trend of increasing anti-depressant prescription. Model D was replicated and so was Model C except that the interaction between social class and baseline GHQ in model C was not significant (p=0.115). This unexpected finding suggests that respondents from a non-manual social class were more likely to be successfully managing their distress with medication than respondents from a manual class.

Table A6 displays results from models using continuous GHQ scores. These required the modal latent class to be treated as an observed variable (i.e. a two-step approach) because latent class membership was otherwise unstable to the addition of the age 57 GHQ scores. This caveat aside, similar results were obtained for model C, and in model D the interaction between class and baseline GHQ was reduced by 21.4% but remained significant.

**Table A1: Odds ratios for sleep class membership with and without adjustment for baseline psychiatric distress**

(distress measured without the inclusion of the sleep item)

|  | Model A: Predicting sleep | | Model B: Predicting sleep with adjustment for psychiatric distress | |
| --- | --- | --- | --- | --- |
|  | OR | 95% CI | OR | 95% CI |
| *Episodic Maintenance Class (ref: Healthy)* | | | | |
| Female Gender | 0.85 | (0.49-1.47) | 0.80 | (0.44-1.45) |
| Manual Social Class | 1.61* | (0.92-2.80) | 1.51 | (0.61-3.73) |
| GHQ (2+) at age 36 |  |  | 3.67** | (1.81-7.45) |
| Social Class and GHQ Interaction |  |  | 1.19 | (0.27-5.16) |
| *Developing Class (ref: Healthy)* | | | | |
| Female Gender | 4.20** | (2.68-6.59) | 4.02** | (2.52-6.42) |
| Manual Social Class | 1.86** | (1.27-2.74) | 1.73* | (1.00-2.99) |
| GHQ (2+) at age 36 |  |  | 2.32** | (1.26-4.28) |
| Social Class and GHQ Interaction |  |  | 1.54 | (0.40-5.97) |
| *Chronic Mixed Class (ref: Healthy)* | | | | |
| Female Gender | 1.36 | (0.78-2.36) | 1.15 | (0.63-2.10) |
| Manual Social Class | 3.33** | (1.69-6.57) | 1.10 | (0.37-3.24) |
| GHQ (2+) at age 36 |  |  | 3.02** | (1.32-6.90) |
| Social Class and GHQ Interaction |  |  | 7.92** | (2.16-29.12) |

*p<0.1

**p<0.05

**Table A2: Odds ratios for sleep class membership with and without adjustment for baseline psychiatric distress**

(taking anti-depressant medication is used as an additional indicator of distress)

|  | Model A: Predicting sleep | | Model B: Predicting sleep with adjustment for psychiatric distress | |
| --- | --- | --- | --- | --- |
|  | OR | 95% CI | OR | 95% CI |
| *Episodic Maintenance Class (ref: Healthy)* | | | | |
| Female Gender | 0.85 | (0.49-1.47) | 0.81 | (0.45-1.46) |
| Manual Social Class | 1.59* | (0.92-2.77) | 1.55 | (0.65-3.69) |
| Psychiatric Distress at age 36 |  |  | 3.57** | (1.82-7.02) |
| Social Class and Distress Interaction |  |  | 1.26 | (0.31-5.10) |
| *Developing Class (ref: Healthy)* | | | | |
| Female Gender | 4.21** | (2.68-6.60) | 4.00** | (2.50-6.40) |
| Manual Social Class | 1.86** | (1.27-2.72) | 1.74* | (0.99-3.04) |
| Psychiatric Distress at age 36 |  |  | 2.30** | (1.23-4.29) |
| Social Class and Distress Interaction |  |  | 1.40 | (0.36-5.43) |
| *Chronic Mixed Class (ref: Healthy)* | | | | |
| Female Gender | 1.36 | (0.78-2.37) | 1.09 | (0.59-2.01) |
| Manual Social Class | 3.34** | (1.69-6.57) | 0.90 | (0.26-3.09) |
| Psychiatric Distress at age 36 |  |  | 3.51** | (1.59-7.77) |
| Social Class and Distress Interaction |  |  | 9.23** | (2.39-35.60) |

*p<0.1

**p<0.05

**Table A3: Odds ratios for sleep class membership with and without adjustment for baseline psychiatric distress**

(continuous GHQ scores)

|  | Model A: Predicting sleep | | Model B: Predicting sleep with adjustment for psychiatric distress | |
| --- | --- | --- | --- | --- |
|  | OR | 95% CI | OR | 95% CI |
| *Episodic Maintenance Class (ref: Healthy)* | | | | |
| Female Gender | 0.85 | (0.49-1.47) | 0.76 | (0.42-1.37) |
| Manual Social Class | 1.60* | (0.92-2.78) | 1.47 | (0.60-3.62) |
| GHQ score at age 36 |  |  | 1.29** | (1.14-1.47) |
| Social Class and GHQ Interaction |  |  | 1.20 | (0.69-2.07) |
| *Developing Class (ref: Healthy)* | | | | |
| Female Gender | 4.20** | (2.68-6.60) | 3.88** | (2.42-6.22) |
| Manual Social Class | 1.85** | (1.26-2.72) | 1.45 | (0.78-2.67) |
| GHQ score at age 36 |  |  | 1.14** | (1.01-1.28) |
| Social Class and GHQ Interaction |  |  | 1.31 | (0.80-2.14) |
| *Chronic Mixed Class (ref: Healthy)* | | | | |
| Female Gender | 1.36 | (0.78-2.37) | 1.07 | (0.63-1.81) |
| Manual Social Class | 3.35** | (1.70-6.61) | 1.09 | (0.45-2.67) |
| GHQ score at age 36 |  |  | 1.27** | (1.11-1.44) |
| Social Class and GHQ Interaction |  |  | 1.65** | (1.03-2.66) |

*p<0.1

**p<0.05

**Table A4: Odds ratios for psychiatric distress at age 57, with and without adjustment for insomnia symptoms**

(distress is measured without the inclusion of the sleep item)

|  | Model C: Predicting distress | | Model D: Predicting distress with adjustment for sleep | |
| --- | --- | --- | --- | --- |
|  | OR | 95% CI | OR | 95% CI |
| Female Gender (ref: Male) | 1.45* | (0.98-2.15) | 1.23 | (0.76-1.99) |
| Manual Social Class  (ref: Non-Manual) | 1.08 | (0.60-1.95) | 0.96 | (0.50-1.84) |
| GHQ (2+) at age 36 (ref: <2) | 2.23** | (1.45-3.43) | 1.78** | (1.12-2.81) |
| Gender and Social Class Interaction | 2.11* | (0.98-4.59) | 2.28** | (1.05-4.92) |
| Social Class and GHQ Interaction | 1.99* | (0.94-4.22) | 1.42 | (0.66-3.08) |
| Episodic Maintenance Class  (ref: Healthy) |  |  | 2.65** | (1.22-5.77) |
| Developing Class (ref: Healthy) |  |  | 4.22** | (2.42-7.35) |
| Chronic Mixed Class (ref: Healthy) |  |  | 8.50** | (4.97-14.54) |

*p<0.1

**p<0.05

**Table A5: Odds ratios for psychiatric distress at age 57, with and without adjustment for insomnia symptoms**

(taking anti-depressant medication is used as an additional indicator of distress)

|  | Model C: Predicting distress | | Model D: Predicting distress with adjustment for sleep | |
| --- | --- | --- | --- | --- |
|  | OR | 95% CI | OR | 95% CI |
| Female Gender (ref: Male) | 1.48** | (1.05-2.08) | 1.31 | (0.87-1.98) |
| Manual Social Class  (ref: Non-Manual) | 0.97 | (0.54-1.77) | 0.86 | (0.44-1.68) |
| Psychiatric Distress at age 36 | 2.51** | (1.73-3.65) | 1.96** | (1.34-2.86) |
| Gender and Social Class Interaction | 2.29** | (1.12-4.66) | 2.51** | (1.24-5.11) |
| Social Class and Distress Interaction | 1.80 | (0.87-3.76) | 1.22 | (0.54-2.73) |
| Episodic Maintenance Class  (ref: Healthy) |  |  | 2.80** | (1.38-5.65) |
| Developing Class (ref: Healthy) |  |  | 3.63** | (2.06-6.38) |
| Chronic Mixed Class (ref: Healthy) |  |  | 9.39** | (4.98-17.73) |

*p<0.1

**p<0.05

**Table A6: Model estimates for psychiatric distress at age 57, with and without adjustment for insomnia symptoms**

(continuous GHQ scores)

|  | Model C: Predicting distress | | Model D: Predicting distress with adjustment for sleep | |
| --- | --- | --- | --- | --- |
|  | Beta | Standard Error | Beta | Standard Error |
| Female Gender (ref: Male) | 0.339* | 0.189 | 0.163 | 0.192 |
| Manual Social Class  (ref: Non-Manual) | -0.029 | 0.277 | -0.214 | 0.266 |
| GHQ score at age 36 | 0.168** | 0.049 | 0.126** | 0.045 |
| Gender and Social Class Interaction | 0.887* | 0.516 | 0.942** | 0.470 |
| Social Class and GHQ Interaction | 0.345** | 0.084 | 0.271 | 0.078 |
| Episodic Maintenance Class  (ref: Healthy) |  |  | 1.039** | 0.300 |
| Developing Class (ref: Healthy) |  |  | 1.355** | 0.237 |
| Chronic Mixed Class (ref: Healthy) |  |  | 2.156** | 0.405 |

*p<0.1

**p<0.05
